# Antibiotic stewardship in Indian palliative care: a single-center retrospective study

**DOI:** 10.1017/ash.2023.468

**Published:** 2023-11-08

**Authors:** David Thomas, Vijayvardhan Kamalumpundi, Amirtha Thampi, Kashelle Lockman, Mary B. Carter, Navjyot Vidwan, Ann Broderick

**Affiliations:** 1 University of Louisville, School of Medicine, Louisville, KY, USA; 2 Department of Medicine, University of Minnesota, Minneapolis, MN, USA; 3 Roy J. and Lucille A. Carver College of Medicine, Iowa City, IA, USA; 4 Pallium India, Thiruvananthapuram, Kerala, India; 5 University of Iowa, College of Pharmacy, Iowa City, IA, USA; 6 Iowa City VA Medical Center, Iowa City, IA, USA

## Abstract

**Objective:**

Characterize antibiotic prescribing behaviors at an Indian palliative care center after the initiation of the Antibiotic Order Form (AOF): an antibiotic stewardship program involving a paper form to track antibiotic use and to provide prescription guidelines.

**Design:**

Retrospective chart review.

**Setting:**

Trivandrum Institute of Palliative Sciences (TIPS) is a palliative care organization in Kerala, India.

**Methods:**

Antibiotic prescription data and patient data were collected for adult patients treated at TIPS between January 1, 2017, and October 31, 2019. Descriptive statistics and a Zero-Inflated Poisson regression model were used to analyze antibiotic prescriptions. AOF completion and prescription concordance with institutional guidelines were also evaluated.

**Results:**

Out of 7,450 unique patients, 675 (9%) were prescribed 1,448 antibiotics. Age was the strongest factor in determining the number of antibiotic courses with each additional year of age decreasing the expected antibiotic prescription count by 2% per year. The most common antibiotics prescribed were topical metronidazole (44%) and penicillins (29%). Among patients who died, 5% were prescribed antibiotics within the final month of life. In total, 32% of antibiotic prescriptions were documented in AOFs, and 18% were concordant with all institutional antibiotic prescribing guidelines.

**Conclusions:**

This study is the first to analyze an antibiotic stewardship intervention in a palliative care setting within a low- and middle-income country. This retrospective study provides a benchmark of antibiotic use within Indian palliative care and highlights areas for future stewardship research including topical metronidazole use within palliative care and higher rates of antibiotic use among younger palliative care patients.

## Introduction

Antibiotic use among patients receiving palliative and hospice care is a global challenge. When addressing infection among palliative and hospice patients, providers are faced with a dilemma. The challenges include concurrently managing symptoms from possible infection, minimizing the incidence of drug resistance, negotiating an emotionally charged atmosphere, and following the patient’s goals of care. These often conflicting priorities might result in empiric antibiotic use. Yet, there are no established guidelines describing how providers should navigate this clinical setting, and, consequently, antibiotic use varies widely across palliative and hospice care settings.

A systematic review of palliative and hospice care research across several countries revealed that 4% to 84% of patients in palliative or hospice care receive antibiotics.^1^ Among patients in the final weeks of life, several studies suggest that at least one-quarter or more of patients receive at least one antibiotic – often in the absence of a documented infection and despite discussions regarding the appropriateness of antibiotic use.^2–7^ The varied use of antibiotics across care settings raises concerns about antibiotic resistance and underscores the need for antibiotic stewardship within palliative and hospice care. Antibiotic stewardship programs (ASPs) are a proven avenue for safely standardizing antibiotic use while improving patient outcomes across multiple clinical settings.^8^ However, little has been explored regarding the utility and design of ASPs in palliative and hospice care, and no studies to date have attempted to explore the use of antibiotics or ASPs among palliative care physicians in low- and middle-income countries (LMIC). Given that palliative care research is predominantly focused on Europe and North America, there is a pressing need to explore the LMIC palliative care experience, especially in regard to antibiotic stewardship.^9^

This study addresses this gap in the literature with a retrospective analysis of records from an Indian palliative care center, Trivandrum Institute of Palliative Sciences (TIPS), following the introduction of an ASP. This study identifies crucial patterns of antibiotic use and suggests potential avenues to support antibiotic stewardship within an LMIC palliative care setting.

## Methods

### Setting

TIPS is the demonstration site for Pallium India, a charitable organization involved in palliative care education and patient care. Located in Kerala, India, TIPS provides care to inpatients in a 13–15 bed unit, to outpatients in clinics, and in their home-based program to any patient with life-limiting illness. During the time of the study, TIPS cared for approximately 3,300 patients annually (300 inpatient, 1,500 outpatient, and 1,500 home care) with 6–7 physicians and an annual mortality of 25%–30%. The TIPS patient population includes patients with terminal illnesses such as end-stage cancer as well as patients with chronic care needs such as chronic kidney disease and diabetes. At the time of this study, TIPS used paper clinical charting; however, patient registration, death data, and pharmacy records were recorded in encrypted Microsoft Excel spreadsheets.

In December 2016, TIPS leadership launched an internal review to evaluate antibiotic prescribing practices at TIPS. This preliminary review revealed that antibiotic prescriptions were inconsistently documented. Moreover, TIPS physicians did not have access to standardized institutional guidelines when prescribing antibiotics. As a result, TIPS, in partnership with the University of Iowa, instituted an ASP in January 2017 with a goal to establish institutional guidelines for antibiotic use and to document antibiotic use at TIPS.

Traditionally, all medications prescribed by TIPS physicians are dispensed by a central pharmacy at TIPS headquarters. Because care teams manage patients across multiple care settings, the TIPS pharmacy dispenses a pre-allotted stock of medications to each care team as well as a Medicine Replacing Book: a standardized paper pad that documents all prescriptions dispensed to patients. When prescribing a medication, TIPS physicians document the medication in both the patient chart as well as a Medicine Replacing Book and dispense the medication directly to the patient. Care teams then return to TIPS each day, submit the Medicine Replacing Book to the pharmacy, and TIPS pharmacists replenish stocks of any dispensed medications.

### Intervention

The novel antibiotic stewardship program was initiated with an interprofessional committee of physicians, nurses, and pharmacists, who designed an additional paper form known as the antibiotic order form (AOF, Supplementary File 1a and 1b). The AOF contained institutional antibiotic prescribing guidelines on one side and a template for tracking antibiotic prescriptions on the other side. When prescribing antibiotics (topical or systemic), TIPS physicians were expected to review the prescribing guidelines, dispense a guideline-concordant antibiotic, and submit the completed AOF alongside their Medication Replacing Book to the TIPS pharmacy at the end of each day. Antibiotic order forms were collated by pharmacy staff and reviewed monthly by the antibiotic stewardship committee. The antibiotic stewardship committee was led by a local champion [AT] who chaired committee discussions regarding antibiotic use and piloted changes to institutional guidelines. All TIPS clinical staff were initially educated regarding AOFs during monthly administrative staff meetings and then reminded annually to continue AOF use. The AOF template and institutional guidelines were updated once by the antibiotic stewardship committee in March 2018 to include multiple changes such as the removal of fluoroquinolones to reflect changes in resistance at local hospitals as well as the addition of topical metronidazole for wound odor management (Supplementary File 2a and 2b).

### Design

This single-center, retrospective study was approved by the Institutional Review Board of the University of Louisville (IRB 18.0716) and the Pallium India Institutional Ethics Committee (IEC-11/2018). Data included antibiotic prescription data and identifiable patient data for all adult patients (≥18 years of age) prescribed antibiotics by TIPS medical staff between January 1, 2017, and October 31, 2019. The investigator [DT] collated antibiotic prescription data from the available records into an encrypted spreadsheet. Available prescription data did not stratify by care setting (inpatient, outpatient, and home care), so care setting was not collected. Patient data for antibiotic recipients were abstracted from available TIPS databases including TIPS patient death registry, TIPS patient palliative care registry, and daily clinic logs. All data handling was completed under the supervision of the local TIPS investigator and antibiotic stewardship committee local champion [AT].

### Statistical analysis

Planned study analysis included descriptive statistics of antibiotic prescription counts, AOF use, and guideline concordance as well as regression analysis of antibiotic prescription counts. However, data such as antibiotic dosage and duration data were available for only a minority of antibiotic prescriptions. Therefore, to evaluate for trends in antibiotic use, patients were categorized into 2 groups: (1) patients who received only 1 antibiotic prescription during the study period; and (2) patients who received 2 or more antibiotic prescriptions during the study period. Patient grouping by number of antibiotic prescriptions was used to compare antibiotic use between patients and to assess if there were target populations for future stewardship intervention. Data was modeled using a Zero-Inflated Poisson regression model. This is a mixture model with a binomial part and a Poisson part that assumes the data contains 2 types of patients: a group receiving one prescription and a group receiving more than 1 prescription where most patients fall into 1 group.^10,11^ This multivariate Poisson analysis was used to model gender, diagnosis, and age with the number of antibiotics prescribed. Statistical analysis was performed in RStudio (Posit, Boston, MA, USA), and the alpha threshold was set at 0.05.

Of note, patient age refers to the age at the time of registration for palliative care and not the age at the time when receiving antibiotics. Diagnosis refers to the diagnosis that qualifies a patient for palliative care. In order to attain adequately sized diagnosis groupings for the regression model, diagnoses were organized into chronic disease and non-chronic disease categories (Supplementary File 3).

Antibiotic stewardship program concordance was evaluated based on AOF completion and concordance of antibiotic prescriptions with institutional antibiotic prescribing guidelines. Completion refers to the percentage of total antibiotic prescriptions (as documented in the Medicine Replacement Book) that were documented on AOFs. Concordance was assessed according to 4 criteria: antibiotic choice, dose, frequency, and duration in days. Antibiotic dose, frequency, and duration were considered concordant only if the antibiotic choice reflected institutional antibiotic prescribing guidelines. Guidelines were available only for urinary tract infections, wound infections, and respiratory infections. Therefore, analysis of concordance was limited to AOFs indicating 1 of those 3 infection types. Because the AOF template and guidelines were updated in March 2018, analysis of concordance data was completed based on the prescribing guidelines listed on each AOF at the time of use. Prescription data collected from other sources such as Medication Replacement Books do not list indications for antibiotic use and were excluded from concordance analysis.

## Results

**Figure 1.**
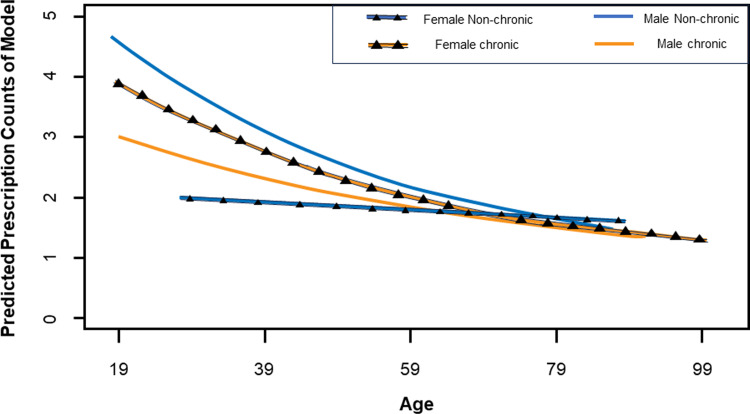
Predicted prescription counts across age. Zero-inflated Poisson regression model showing predicted antibiotic prescription counts based on gender, diagnosis type, and age.

Out of 7,450 patients treated by TIPS staff during the study period, 675 (9%) patients received antibiotics with 1,448 total antibiotic courses prescribed. Patient gender and diagnosis data for antibiotic recipients are categorized in Table 1. The median age at palliative care registration for all antibiotic recipients was 60 years [IQR 20]. In the binomial analysis, each additional year of age decreased the expected antibiotic prescription count by 2% per year (*P* < 0.001, Table 2). The accompanying graph (Figure 1) shows how diagnosis category and gender are represented across ages in the Zero-Inflated Poisson regression model. Diagnosis distribution within the groups, particularly the non-chronic disease group, varied by gender and was not able to be accounted for in this model; however, the disparity is accurately represented. The multivariate Poisson analysis confirmed that age is the most significant variable affecting the number of antibiotic courses prescribed and is more striking for men than women.

**Table 1.** Baseline characteristics of patients prescribed an antibiotic

Diagnosis	Antibiotic recipient patient count	Male	Median age in years (IQR)	Percent treated at final month of life	Median number of antibiotics (IQR)	Patients receiving 1 antibiotic	Patients receiving >2 antibiotics	Total antibiotic prescriptions
Chronic disease	277	137 (49%)	65 (21)	53 (19%)	1 (1)	178 (64%)	99 (35%)	517 (36%)
Non-chronic disease	398	231 (58%)	56 (20)	80 (20%)	1 (1)	235 (59%)	163 (41%)	931 (64%)
Total	675	386 (55%)	60 (20)	133 (20%)	1 (1)	413 (61%)	262 (39%)	1448

Listed percentages for patient counts were calculated as percentages of antibiotic recipient patient count for each row. Listed percentage for total antibiotic prescriptions is percentage of column total.

**Table 2.** Parameter estimates for zero-inflated Poisson regression model

	Estimate	Standard error	*P* value
Binomial			
Intercept	0.27	0.09	<0.001
Age	0.02	0.01	<0.001
Poisson			
Intercept	0.65	0.11	<0.001
Age	0.00	0.01	0.65
Gender-male	0.35	0.13	0.01
Chronic disease	0.21	0.15	0.16
Age, gender-male, and chronic disease	0.03	0.01	0.04

### Antibiotic prescriptions by antibiotic class

**Figure 2.**
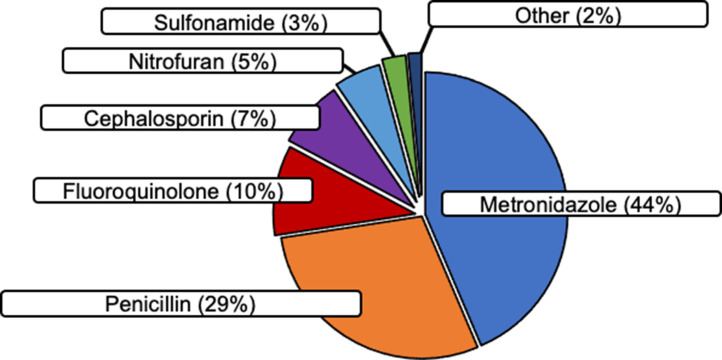
Antibiotics prescribed. Pie chart documenting the distribution of all antibiotic prescriptions by antibiotic class at the Trivandrum Institute of Palliative Sciences between January 1, 2017, and October 31, 2019.

Overall, the most common antibiotics prescribed were topical metronidazole (44% of all prescriptions, Figure 2) and penicillins (29%). Topical metronidazole was used to control wound odor, a common concern with malignant wounds and pressure sores. Following the March 2018 revision of TIPS guidelines, fluoroquinolones were removed from the TIPS formulary with an observed decrease in annual fluoroquinolone use in 2018 and 2019 (Table 3).

**Table 3.** Antibiotic distribution and guideline concordance

Antibiotic class	Annual total prescriptions: 2017	Annual total prescriptions: 2018	Annual total prescriptions: 2019^1^	Number of prescriptions charted on AOFs^2^	Concordant with antibiotic choice (number of prescriptions)^3^	Concordant with all ASP guidelines (number of prescriptions)^3^
Sulfonamide	0 (0%)	34 (9%)	11 (2%)	40 (89%)	37 (93%)	5 (13%)
Nitrofurantoin	18 (4%)	41 (10%)	18 (3%)	55 (71%)	54 (100%)	22 (41%)
Penicillins	93 (19%)	154 (39%)	173 (31%)	232 (55%)	145 (76%)	30 (16%)
Fluoroquinolones	115 (23%)	28 (7%)	5 (1%)	50 (34%)	0	0
Cephalosporins	59 (12%)	29 (7%)	17 (3%)	36 (34%)	16 (59%)	5 (19%)
Metronidazole	211 (42%)	95 (24%)	325 (59%)	29 (5%)	8 (53%)	–
Other^4^	6 (1%)	11 (3%)	5 (1%)	16 (73%)	–	–
Total	502	392	554	458	–	–

1 Only the first 10 months of 2019 were analyzed.

2 The percentage was calculated based on the number of prescriptions charted on AOFs for an antibiotic class divided by the total number of prescriptions for an antibiotic class.

3 Concordance was evaluated for Antibiotic Order Form prescriptions listing an indication of urinary tract infection, wound infection or respiratory infection. Concordance percentages were calculated by dividing the number of antibiotics concordant with antibiotic choice or concordant with all antibiotic stewardship guidelines by the total number of prescriptions per antibiotic class for the aforementioned indications. “–” are inserted if guidelines on choice, dosage, frequency or duration were not available for that class.

4 Antibiotics in the “other” category were not included in guideline concordance data. Abbreviations: AOF, antibiotic order form; ASPs, antibiotic stewardship practices.

### Antibiotic prescriptions during the final month of life

During the study period, 2,872 (39%) TIPS patients died. Among 675 antibiotic recipients, 282 (42%) died with 133 (47% of deceased antibiotic recipients and 5% of all deceased patients) receiving antibiotics within the final month of life. Since data from deceased patients who did not receive antibiotics were not collected, we were unable to determine which demographic or clinical factors predicted antibiotic use in the final month of life.

### Antibiotic order form completion

**Figure 3.**
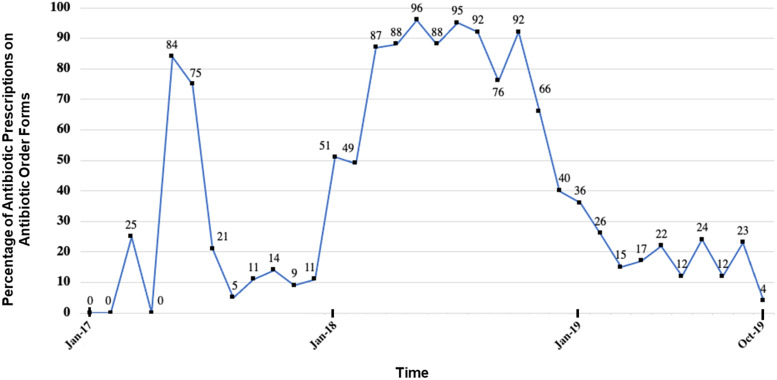
Monthly Antibiotic Order Form completion. Line chart detailing monthly percentage of antibiotic prescriptions documented on Antibiotic Order Forms.

In total, 458 (32%) of all antibiotic prescriptions were documented in AOFs. Topical metronidazole was the least documented antibiotic with only 5% of prescriptions listed on AOFs. Sulfonamides were the most documented class with 89% listed on AOFs (Table 3). Monthly AOF completion ranged between 0% and 96%. The highest levels of monthly AOF completion were between March and November 2018 with more than 60% of all antibiotic prescriptions documented in AOFs across 8 consecutive months (Figure 3). Due to limitations in TIPS staff access during the 2019 Coronavirus pandemic, factors affecting variable AOF completion were not assessed for the study period.

### Antibiotic order form concordance with institutional prescribing guidelines

A total of 386 AOFs (84% of all AOFs and 27% of all prescriptions) met the criteria for analyzing prescription concordance with institutional prescribing guidelines. Of these, 260 (67%) reflected the guideline-recommended antibiotic choice and 70 (18%) demonstrated concordance across all 4 criteria of antibiotic choice, dose, frequency, and duration. Notably, there was an observed increase in concordance of antibiotic choice from 31% of AOFs in 2017 to 78% and 76% in 2018 and 2019, respectively.

## Discussion

To our knowledge, this is the first study to explore how physicians prescribe antibiotics and adhere to antibiotic stewardship guidelines within a low- and middle-income country palliative care setting. The results of this study demonstrate that: (1) younger patients were more likely to receive more than one antibiotic course; (2) nearly half of all deceased antibiotic recipients received antibiotics within the final month of life; and (3) physicians did not consistently complete ASP documentation or follow institutional guidelines when prescribing antibiotics. Moreover, the focus on antibiotic stewardship led to early changes to antibiotic prescribing behaviors at TIPS including fluoroquinolone removal based on regional antibiotic resistance, recognition of variability in prescribing practices, and increased awareness of high topical metronidazole use for the management of wound odor. These results provide a benchmark of antibiotic use and stewardship concordance within an LMIC palliative care setting and highlight avenues for optimizing future antibiotic stewardship interventions.

Regarding patient age, younger age appears to be the strongest factor affecting antibiotic prescribing. Age is a known factor affecting access to palliative care therapies with previous studies suggesting that younger age may independently influence palliative care physician perception of patient needs, length of palliative care stay, and access to life-prolonging therapies.^12,13^ Moreover, younger palliative care patients within LMICs may have a higher incidence of comorbidities such as debilitating traumatic injury, which may increase infection risk and lead to higher antibiotic use.^14–16^ Due to our limited sample size, multivariate analysis of sub-groups such as traumatic injury patients was not possible. Nevertheless, the recognition of higher antibiotic use among younger patients provides a target for future antibiotic stewardship research and intervention.

In terms of antibiotic choice, the use of penicillins at TIPS (29% of all antibiotics) is comparable to usage rates published by other medical centers in India. A study in Puducherry documented that amoxicillin accounts for ≥30% of antibiotic prescriptions across primary and tertiary care centers in the area.^17^ Similarly, a survey of antibiotic usage among 16 hospitals across India showed that penicillins with beta-lactamase inhibitors were the most frequently prescribed antibiotics, accounting for 47.6% of antibiotic prescriptions.^18^ Yet, penicillin use at TIPS is rising, and increased antibiotic use is a known driver of resistance. Given the heavy reliance of TIPS and other Indian institutions on penicillins, optimizing antibiotic surveillance will be crucial to capturing excessive penicillin use and curbing future resistance.

Likewise, topical metronidazole use at TIPS (44% of all antibiotics) may be comparable to other facilities in India and internationally. Metronidazole is exclusively used as a topical agent at TIPS for the management of wound odor. However, due to cost restrictions, topical metronidazole at TIPS is often compounded by nurses on-site by crushing metronidazole tablets into petroleum jelly. This is a widely recommended method for palliative care teams to control wound odor, but, without a standardized formulation, drug concentrations may vary between patients.^19,20^ This nursing-led intervention likely complicated antibiotic stewardship documentation and may account for the reduced number of metronidazole prescriptions recorded on AOFs. Considering that the overuse of topical antibiotics may be also linked to increasing antibiotic resistance, tracking and standardizing formulations of topical metronidazole may be another priority for future stewardship initiatives within palliative and hospice care.^21^

Regarding antibiotic use at the end of life, 47% of antibiotic recipients who died received antibiotics in the final month of life. While this occurred in only 5% of all TIPS patients who died, it is appropriate to review the reasons for antibiotics in this setting, both at the bedside and in the antibiotic stewardship literature. Providers may be attempting to prolong survival, fulfill patient goals of care, or alleviate patient discomfort. A 2020 scoping review suggests that antibiotics do provide a limited survival benefit to some patients nearing the end of life and may also mitigate symptoms associated with certain infections such as urinary tract infections; however, these benefits may come at the cost of prolonging the dying process, compounding adverse effects, and promoting antibiotic resistance.^22^ Existing stewardship guidelines – including AOF guidelines – do not typically reflect the challenges surrounding prescription decisions at the end of life, ultimately limiting their use among terminally ill patients. Therefore, a focus for future stewardship efforts may be to develop guidance to help providers navigate discussions surrounding antibiotic use at the end of life. Instead of strict prescription guidelines, it may be more appropriate for ASPs to provide a model for shared clinical decision-making that integrates patient preferences into existing guidelines to create an individualized care plan.^23^

Finally, completion of ASP documentation and concordance of antibiotic prescriptions with institutional antibiotic prescribing guidelines were inconsistent and revealed at least 1 potential area for improvement: leadership engagement. Specifically, engagement of clinical leadership appeared to increase use of AOFs. From January through March 2018, TIPS antibiotic stewardship committee members convened to update existing institutional antibiotic prescribing guidelines due to increasing concerns regarding local fluoroquinolone resistance. There was a marked increase in AOF use during subsequent months and an increase in concordance of antibiotic choice during subsequent years. These results suggest that even inconsistent implementation of an ASP with invested clinical leadership may provide an accessible avenue to introduce antibiotic stewardship into the LMIC palliative care setting with measurable improvements in antibiotic utilization.

This study has several limitations. Several of the factors that may affect physician prescribing behavior including patient comorbidities, bacterial culture data, symptom burden before and after antibiotic use, socioeconomic status, location (inpatient, outpatient, and home care), and length of stay on the inpatient unit were not available for analysis. Additionally, due to severe flooding in 2018, some paper records may have been lost prior to data collection, thus limiting available results. Lastly, the scope of this study was restricted to prescribing behaviors by TIPS medical staff and may not necessarily reflect actual antibiotic consumption by patients.

Despite these limitations, this retrospective study is the first to assess the outcomes of a recently launched interprofessional antibiotic stewardship program in an Indian palliative care setting. Results from this study fill a gap in the literature and could potentially help design future prospective studies using the AOF at other healthcare centers. Based on our findings, TIPS officials plan to further evaluate metronidazole use, increase awareness of the need for accurate record keeping by site of care, implement regular review of the AOF, and educate physicians on the consistent use of recommended antibiotic choices, dose, and duration. Further investigation is needed to evaluate perspectives of palliative care physicians, pharmacists, and nurses regarding antibiotic stewardship as well as factors that predict antibiotic use in the final month of life.

## Supporting information

Thomas et al. supplementary material 1 Thomas et al. supplementary material

Thomas et al. supplementary material 2 Thomas et al. supplementary material

Thomas et al. supplementary material 3 Thomas et al. supplementary material

## Supplementary material

The supplementary material for this article can be found at https://doi.org/10.1017/ash.2023.468
