# Supplementary material for: Antibiotic stewardship in Indian palliative care: a single-center retrospective study
Source: Antimicrob Steward Healthc Epidemiol. 2023 Nov 8;3(1):e196. doi: 10.1017/ash.2023.468 (PMC10654949; doi:10.1017/ash.2023.468)
Supplement: Thomas et al. supplementary material 1 — Thomas et al. supplementary material [file S2732494X23004680sup001.docx]

**Supplementary File 1a: Antibiotic Order Form Front**

| **Date:** |  |
| --- | --- |
| **Patient ID:** |  |

**Antibiotic Order Form**

Pallium India

| **Indication Details:** |  |  |  |  |  |  |
| --- | --- | --- | --- | --- | --- | --- |
| **Indication (check one)** | CAUTI | UTI | Upper Respiratory | Wound | Other: |  |
|  |  |  |  |  |  |  |
| **Suspected or Confirmed?** |  | Suspected |  | Confirmed |  |  |
|  |  |  |  |  |  |  |
| **Cultures Ordered?** |  | Yes |  | No |  |  |

| **Infection Details:** |  |  |  |  |  |  |
| --- | --- | --- | --- | --- | --- | --- |
| **Site of Infection:** |  |  |  |  |  |  |
|  |  |  |  |  |  |  |
|  |  |  |  |  |  |  |
| **Signs & Symptoms?** | Fever | Breathlessness | Dysuria | Redness/Swelling | Diarrhea |  |
|  |  |  |  |  |  |  |
|  | Other: |  |  |  |  |  |

| **Treatment Details:** | |  |  |  |  |  |  |
| --- | --- | --- | --- | --- | --- | --- | --- |
|  | |  |  |  |  |  |  |
| **Choice of antibiotic:** |  | |  |  |  |  |  |
| **Treatment duration:** |  | |  |  |  |  |  |
|  |  | |  |  |  |  |  |
|  |  | |  |  |  |  |  |
| **Antibiotics provided:** | None | | 1-day supply | 2-day supply | Full treatment course | Other: |  |
|  |  | |  |  |  |  |  |
|  |  | |  |  |  |  |  |
| **Prescription provided** | None | |  | Full treatment duration provided |  | Other: |  |
|  |  | |  |  |  |  |  |
|  |  | |  |  |  |  |  |
| **Education provided:** | Signs of allergic reaction | | | Recommendation of course completion  (Even if patient feels better) | |  |  |
|  |  | |  |  |  |  |  |
|  | Explanation of antibiotic resistance | | | Other: | |  |  |

**Supplementary File 1b: Antibiotic Order Form Back**

**Respiratory tract infections**

| CONDITION | CAUSE | ANTIBIOTICS | COMMENTS |
| --- | --- | --- | --- |
| Acute pharyngitis- “sore throat” | Viral | None required | Most adults with acute pharyngitis have an illness that will get better with saline gargles, antipyretics, and analgesics |
| Strep throat | Group A streptococcus | Amoxicillin 500mg TID for 10 days | Give only if fever, tonsillar exudates, no cough, and anterior cervical lymphadenopathy |
| Acute bronchitis | Viral | None | - |
| Acute bacterial rhinosinusitis (antibiotics if symptoms persists for 7-10 days, facial pain, and purulent discharge) | Viral | Amoxicillin 500 mg TID for 10 days | - |
| COPD exacerbation (increased sputum, increased color in sputum, increased dyspnea in a smoker/long term smoke exposure) | Strep pneumoniae  H. influenzae  M. catarrhalis | Amoxicillin 500mg TID for 7 days OR doxycycline 100mg BID for 7 days OR Azithromycin 500mg daily for 3 days | - |
| Pneumonia (fever, cough, sputum, lung findings) |  | **Outpatient treatment:**  Azithromycin 500mg for 5 days OR  Doxycycline 100mg BID for 7 days OR  Moxifloxacin 400mg for 5 days  **Inpatient treatment:**  Cefoperazone/sulbactam if ill OR outpatient therapies | - |

**Urinary tract infections**

| CONDITION | CAUSE | Treatment- *adjust when culture results are known* | COMMENTS |
| --- | --- | --- | --- |
| Acute cystitis | - | Nitrofurantoin 100mg BID for 7 days  Norfloxacin 400mg BID for 5 days |  |
| Pyelonephritis (fever and back pain) | - | Amikacin 15mg/kg IV every 24 hours for 7 days for mild illness or 14 days if severe illness |  |
| Catheter associated UTI  Do not check urine unless there is fever, flank pain, delirium and for spinal cord injury patients, hyperreflexia or drastic clinical change.  Bacteria and WBCs in urine + above symptoms= UTI | - | Treat empirically as above until results are known. | Do not take urine from bag. Replace catheter and obtain first urine for culture and sensitivity.  Use Intermittent catheterization/condom cath when infection is resolved.  **Bacteria and WBCs in urine without symptoms is not a UTI. Cloudy urine appearance and foul smell is not a UTI. |

**Skin infections**

| CONDITION | CAUSE | TREATMENT | COMMENTS |
| --- | --- | --- | --- |
| Cellulitis | Staph aureus | Amoxicillin-Clavulanate 500mg every 12 hours +/- Clindamycin 600mg QID |  |
| Deep pressure ulcer | Multiple | Cefoperazone/  Sulbactam +/- Clindamycin 600mg QID | Obtain deep culture and direct therapy accordingly |
| Diabetic foot ulcer | DO NOT START ANTIBIOTICS UNLESS CELLULITIS OR SYSTEMIC INFECTION PRESENT | Need X-Rays to assess for osteomyelitis and arterial doppler | Obtain deep culture |
