# Supplementary material for: Antibiotic stewardship in Indian palliative care: a single-center retrospective study
Source: Antimicrob Steward Healthc Epidemiol. 2023 Nov 8;3(1):e196. doi: 10.1017/ash.2023.468 (PMC10654949; doi:10.1017/ash.2023.468)
Supplement: Thomas et al. supplementary material 2 — Thomas et al. supplementary material [file S2732494X23004680sup002.docx]

**Supplementary File 2a: Updated Antibiotic Order Form Front**

| **🞎 Inpatient**  **🞎 Outpatient**  **🞎 Home visit: site _________** | | **Antibiotic Order Form**  Pallium India | | **Date:**  **Patient Name:**  **Patient ID:** | |
| --- | --- | --- | --- | --- | --- |
| **TO BE COMPLETED BY PHYSICIAN OR NURSE. Deposit completed forms in the pharmacy** | | | | | |
| **Indication Details:** | | | | | |
| **Indication (check one):** | **🞎**  **CAUTI** | **🞎**  **UTI** | **🞎**  **Respiratory Tract** | **🞎**  **Wound** | **🞎**  **Other** |
| **Suspected or Confirmed?** | | **🞎 SUSPECTED** | | **🞎 CONFIRMED** | |
| **Cultures Ordered?** | | **🞎 YES** | | **🞎 NO** | |

| **Infection Details** | | | | | | | | |
| --- | --- | --- | --- | --- | --- | --- | --- | --- |
| **Site of Infection:** | | | **________________________________________________________** | | | | | |
| **Signs & Symptoms:** | **🞎**  **Fever** | **🞎**  **Breathlessness** | | **🞎**  **Cough** | **🞎**  **Dysuria** | **🞎**  **Redness/Swelling** | **🞎**  **Diarrhea** | **🞎**  **Other** |

| **Treatment Details** | **🞎 Initiation of antibiotic** |
| --- | --- |
|  | **🞎 Continuation of antibiotic prescribed outside of Pallium** |
|  | **🞎 Changing to new antibiotic** |

| **Choice of Antibiotic:** | **________________________________________________________** | |
| --- | --- | --- |
| **Treatment Duration:** |  | |
| **Antibiotics provided:** | **🞎 Full treatment course** | |
|  | **🞎 Partial Treatment course: ____ days supply** | |
|  | **🞎 No prescription provided (outpatient/home care only)** | |
| **Education Provided:** | **🞎 Side Effects** | **🞎 Explanation of antibiotic resistance** |
|  | **🞎 Signs of allergic reaction** | **🞎 Other:** |

**Supplementary File 2b: Updated Antibiotic Order Form Back**

**Respiratory Tract Infections**

| CONDITION | LIKELY CAUSE | ANTIBIOTICS | COMMENTS |
| --- | --- | --- | --- |
| Acute Pharyngitis: “sore throat” | Viral | No antibiotics required | Recommend saline gargles, antipyretics and analgesics |
| Strep Throat: If at least 3 of the following: fever, oropharyngeal exudates, anterior cervical lymphadenopathy and no cough | Group A streptococcus | Amoxicillin 500 mg TID for 10 days |  |
| Acute Bronchitis | Viral | No antibiotics |  |
| Acute Bacterial Rhino-Sinusitis: facial pain and purulent drainage | Viral or bacterial | If symptoms < 7-10 days – likely viral: **NO ANTIBIOTICS** required  If persistent symptoms > 7-10 days – suspect bacterial: Amoxicillin-clavulanate 625mg q8h for 5 days | If allergic to penicillin give moxifloxacin 400 mg once daily for 5-7 days ($$$) |
| COPD Exacerbation: Increased sputum, increased color of sputum, increased dyspnea in a smoker/long term smoke exposure | Strep pneumoniae, H. influenzae, M. catarrhalis | Amoxicillin-clavulanate 625mg q8h for 5 days and then re-evaluate if not improved | If allergic to penicillin give moxifloxacin 400 mg once daily for 5-7 days ($$$) |
| Pneumonia: fever, cough, sputum, lung findings | Group A streptococcus | **Outpatient treatment or mild disease**: Amoxicillin-clavulanate 625mg q8h for 5 days  **Inpatient treatment**: Amoxicillin-clavulanate IV 1.2 g q8h for 5-7 days or ceftriaxone IV 2g once daily for 5-7 days transition to oral when possible | If allergic to penicillin give moxifloxacin 400 mg once daily for 5-7 days ($$$) |

**Urinary Tract Infections**

| CONDITION | LIKELY CAUSE | ANTIBIOTICS: adjust when culture results are known | COMMENTS |
| --- | --- | --- | --- |
| Bladder Infection: painful urination, urgency, frequency, nocturia, hematuria, lower abdominal pain | E. coli | Trimethoprim-sulfamethoxazole D5 (cotrimoxazole) 160/800 BID for 5 days * do not use with sulfa allergy | Alternative: nitrofurantoin 100mg BID for 7 days (do not use in patients with reduced renal function e.g. elderly) |
| Kidney Infection: Fever, back pain, nausea, malaise, vomiting | E. coli  K. pneumoniae  P. aeruginosa  Entercoccus | **Mild**: see bladder infection (above)  **Severe:** tazobactam-piperacillin or Cefoperazone-sulbactam for severe illness |  |
| Catheter Associated UTI: Do not check urine unless there is feer, back pain, delirium and for spinal cord injury patients – hyper-reflexia or drastic clinical change. Bacteria and WBCs in urine with above symptoms mean UTI | Multiple | Treat empirically as above until results are known. Treat for 7 days for CAUTI.  Do not take urine from bag. Replace catheter and obtain first urine for culture and sensitivity. | Use intermittent catheterization or condom cath when infection has resolved  Bacteria and WBCs in urine without symptoms is not a UTI  Cloudy appearance and foul smell is not a UTI |

**Skin Infections**

| CONDITION | LIKELY CAUSE | ANTIBIOTICS | COMMENTS |
| --- | --- | --- | --- |
| Cellulitis | Staph aureus | Amoxicillin-Clavulanate 625mg q8h +/- clindamycin 600mg q8h IV for 5 days |  |
| Deep Tissue Infection (Stage 3 or 4) | Multiple | Cefoperazone-sulbactam 3g IV BD +/- clindamycin 600mg IV q8h for 5-7 days | Obtain deep culture and direct therapy (adjust based on renal function) |
| Diabetic Foot Ulcer |  | Need X-ray for osteomyelitis AND arterial doppler | Obtain deep culture: DO NOT START ANTIBIOTICS UNLESS SYSTEMIC INFECTION |
| Malignant Wound Infection: foul-smell, maggot infestation, in presence of cancer | Multiple anaerobes | Remove maggots, metronidazole for wound odor. If fever, treat as above |  |
