# Supplementary material for: Antibiotic stewardship in Indian palliative care: a single-center retrospective study
Source: Antimicrob Steward Healthc Epidemiol. 2023 Nov 8;3(1):e196. doi: 10.1017/ash.2023.468 (PMC10654949; doi:10.1017/ash.2023.468)
Supplement: Thomas et al. supplementary material 3 — Thomas et al. supplementary material [file S2732494X23004680sup003.docx]

**Supplementary File 3: Patient Diagnosis Categories**

TIPS patient diagnoses were categorized into two groups. Specific patient diagnoses are detailed below within the associated group. Notably, patient diagnoses refer only to the diagnosis that qualified a patient for palliative care.

1. Chronic Disease — asthma, chronic obstructive pulmonary disease, chronic kidney disease, filariasis, heart failure, hypertension, liver cirrhosis, lymphedema, muscular dystrophy, neurodegenerative disorders, parkinsonism, peripheral vascular disease, psychiatric illness, rheumatic disease, stroke, type 2 diabetes.

2. Non-Chronic Disease — adenocarcinoma, brain cancer, breast cancer, gastrointestinal cancer, genitourinary cancer, hematologic malignancies, lung cancer, oropharyngeal cancer, skin cancer. traumatic brain injury, traumatic fracture, traumatic hemiparesis, traumatic paraplegia, road traffic accident, spinal cord injury.
